# The Development of Cellular Aging Models Based on AD-MSCs

**DOI:** 10.3390/cells15181634

**Published:** 2026-09-09

**Authors:** Natalia V. Elizova, Ivan V. Zhivodernikov, Vyacheslav S. Vasilyev, Yaroslav D. Tolkachev, Yuliya V. Markina, Alexander M. Markin, Tatiana V. Kirichenko

**Affiliations:** 1 Petrovsky National Research Centre of Surgery, 119435 Moscow, Russiat-gorchakova@mail.ru (T.V.K.); 2 Institute of Plastic Surgery and Cosmetology, 105066 Moscow, Russia

**Keywords:** MSCs, adipose tissue, cellular senescence, SASP, senescence-associated β-galactosidase, fisetin

## Abstract

Cellular senescence of adipose tissue-derived mesenchymal stromal cells (AD-MSCs) is associated with chronic low-grade inflammation, metabolic dysfunction, and impaired adipogenesis contributing to the development of obesity, insulin resistance, and the progression of age-associated diseases. The aim of this study was to develop a model of cellular aging based on AD-MSCs to study the senolytic efficacy of the natural fisetin preparation for the subsequent development of geroprotective therapeutic strategies. The senescent markers SA-β-gal, SASP factors, and cell cycle arrest markers p16, p21, and p53 were assessed in models of replicative and stress-induced aging of AD-MSCs. H_2_O_2_ was identified as the most effective inducer of AD-MSC cellular senescence in comparison with LPS. It has been shown in the model of H_2_O_2_-induced senescence of AD-MSCs that the proportion of SA-β-gal+ cells was 2.0 (0.7)% in non-stimulated AD-MSCs, 81.7 (12.1)% in H_2_O_2_-stimulated AD-MSCs, and 19.1 (5.2)% and 13.0 (3.2)% after short-term (24 h) and long-term (7 days) fisetin treatment, respectively, indicating that fisetin treatment was associated with a lower proportion of SA-β-gal+ cells. Fisetin treatment resulted in a statistically significant decrease in the secretion of MCP-1, especially during long-term incubation. IL-1β showed a significant decrease after 7 days of fisetin treatment compared to H_2_O_2_, but the effect at 24 h was only significant in the group of 7 days of fisetin treatment. Fisetin treatment was also associated with higher p53 and p21 concentrations. Thus, in the present study, a model of H_2_O_2_-induced cellular senescence of AD-MSCs was developed, which can be used to evaluate the geroprotective potential of senotherapeutic preparations; fisetin warrants further evaluation as a potential senotherapeutic agent in this model.

## 1. Introduction

Adipose tissue is a heterogeneous cellular system that includes adipocytes, mesenchymal stromal cells, macrophages and other immune cells, preadipocytes, endothelial cells, pericytes, and fibroblasts [1]. Adipose tissue cells physiologically secrete a wide range of hormones, enzymes, and signaling molecules, which are directly involved in endocrine and immune regulation and metabolism, exerting a direct influence on energy metabolism, insulin sensitivity, and other metabolic functions [2]. A meta-analysis of the studies aiming to reveal the relationship between obesity and aging showed that the prevalence of obesity increases with age, enhancing the risk of hypertension, diabetes mellitus, and coronary heart disease, leading to aging of the organism and premature mortality [3].

Adipose tissue-derived mesenchymal stromal cells (AD-MSCs) provide functional plasticity and maintain adipose tissue homeostasis [4]. Cellular senescence of AD-MSCs, associated with a decrease in proliferative potential and a response to DNA damage caused by factors such as oxidative stress, autophagy impairment, mitochondrial dysfunction, and epigenetic modifications, contributes to the development of the senescence-associated secretory phenotype (SASP) [5], which leads to the formation of the senescent phenotype of AD-MSCs, the development of chronic low-grade inflammation, and impaired adipogenesis. In this regard, the dysfunction of AD-MSCs contributes to the development of obesity, insulin resistance, and the progression of age-associated diseases [6]. In recent years, AD-MSCs have been considered as a potential resource for the development of anti-aging and regenerative therapy strategies [7,8]. In addition, the MSC secretome includes various biomolecules with immunomodulatory, antifibrotic, antiapoptotic, proangiogenic, re-epithelializing, and neurotrophic activity [9].

Currently, the mechanisms of cellular aging are being widely studied with the aim of identifying the most informative biomarkers of senescent cells, developing algorithms for biological age assessment and new senotherapeutic strategies [10]. Given the heterogeneity of cellular senescence, a combined assessment of cell cycle arrest markers, SA-β-gal activity, and SASP components is recommended [11]. The role of p21 in cell cycle arrest and cellular senescence has also been highlighted [12]. The study of the cellular aging of MSCs is relevant due to their widespread involvement in maintaining homeostasis in various tissues, immune regulation, and regenerative potential [13]. Preparations with senolytic and senomorphic effects are a promising and well-studied approach to combating cellular aging, but therapies targeting MSC aging still require further study [14]. Natural preparations based on natural polyphenols deserve special attention. In particular, fisetin is one of the most studied preparations; several pilot clinical trials have demonstrated the effectiveness of fisetin in relation to aging markers with minimal side effects [15]. The aim of this project was to develop a model of cellular aging based on AD-MSCs to identify the most effective biomarkers of cellular aging and to study the senolytic efficacy of the natural fisetin preparation for the subsequent development of strategies for geroprotective therapy.

## 2. Materials and Methods

### 2.1. Isolation of AD-MSCs from Adipose Tissue

To develop a model of cellular aging, 10 mL of adipose tissue was obtained from subcutaneous abdominal fat tissue during a fat removal surgery. For the experimental part of the study, adipose tissue samples were obtained from 8 subjects with a mean age of 37.0 (3.3) years. The non-inclusion criteria were age under 70 years, diabetes mellitus, and the presence of severe chronic diseases and comorbid conditions, such as chronic renal failure, chronic liver failure, and cancer. The study protocol was approved by the local ethics committee of the Petrovsky National Research Centre of Surgery (protocol no. 5; dated 23 May 2025). The adipose tissue was washed under sterile conditions in isotonic phosphate-buffered solution (PBS) twice and centrifuged, removing the bulk of the erythrocytes. After centrifugation, the adipose fraction was diluted with PBS with the addition of type I collagenase at a final concentration of 1 mg/mL. This suspension was incubated in a water bath (37 °C, 45 min). Then, the suspension was diluted with fresh medium in a ratio of 1:1 and centrifuged. The precipitated cells of the stromal–vascular fraction were additionally washed, the remaining fractions were removed, and the cells were centrifuged and cultured for 24 h. Then, the cells were washed to remove non-adherent cells. The cells remaining on the plastic were cultured in the culture medium (DMEM) with low glucose (1 g/L) and 10% FBS, 50 U/mL penicillin, and 50 μg/mL streptomycin under standard conditions (5% CO_2_, 37 °C).

**Figure 1:**
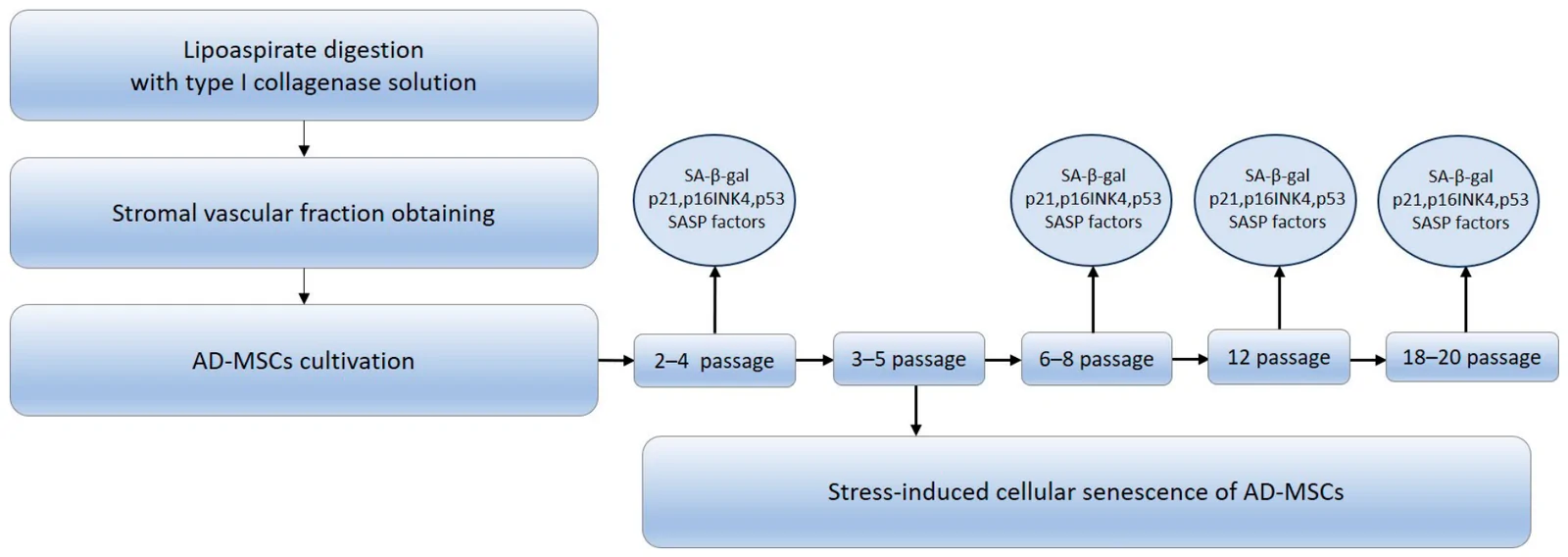
Scheme of isolation and cultivation of AD-MSCs. MSCs, mesenchymal stromal cells; SA-β-gal, senescence-associated β-galactosidase; SASP, senescence-associated secretory phenotype; p21, p16, p53, cell cycle arrest marker proteins.

Cells were cultured in 75 cm^2^ SPL flasks (SPL Lifesciences, Pocheon-si, Korea). Cells were washed with Versene solution (PanEco, Moscow, Russia), scraped from the flask surface with 0.25% trypsin solution (PanEco, Moscow, Russia), and passaged once per week upon reaching 90–95% confluency. The obtained cells were identified as MSCs based on their ability to undergo three-way differentiation in the adipogenic, osteogenic, and chondrogenic directions in accordance with the criteria of the International Society for Cellular Therapy (ISCT) [16]. The isolation and cultivation scheme for AD-MSCs is shown in Figure 1.

### 2.2. Model of Replicative Cellular Senescence

Replicative cellular senescence was assessed in the cultured AD-MSCs. Cell passaging was performed upon reaching 90–95% confluency (once a week), as described above, up to passage 20.

### 2.3. Model of Stress-Induced Cellular Senescence

**Figure 2:**
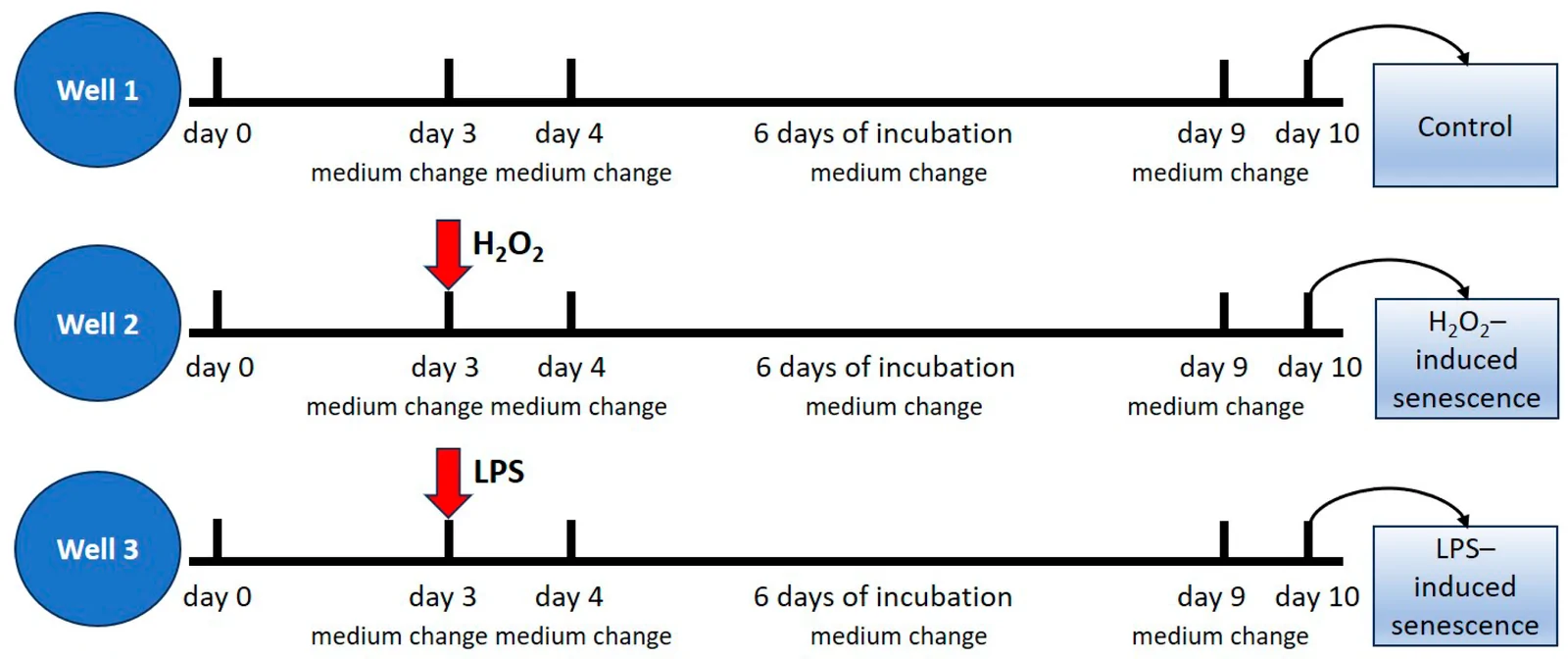
Scheme of the stress-induced cellular senescence model in AD-MSCs.

AD-MSCs were seeded at a density of 1 × 10^4^ cells/cm^2^. Cells were treated when the monolayer reached 70–80% confluence. Lipopolysaccharide (LPS from *Escherichia coli* O111:B4) at a concentration of 1 μg/mL (Sigma-Aldrich, St. Louis, MO, USA) or 50 μM hydrogen peroxide solution (Chimmed, Moscow, Russia) was added in cell culture at day 3 for 24 h to stimulate cellular senescence in 3–4 passages of AD-MSCs. Then, the culture medium was refreshed, and cells were cultured for 7 days before the estimation of senescence markers. A scheme of the stress-induced cellular senescence model is shown at Figure 2.

### 2.4. Analysis of Cell Viability

The cytotoxicity of fisetin and hydrogen peroxide was assessed using the resazurin cell viability assay. Cells were seeded in 96-well plates in a volume of 100 µL. After 48 h, fisetin and hydrogen peroxide were added at various concentrations. After 24 h, the medium was removed, and 100 µL of a resazurin solution (AbiCell Resazurin Cytotoxicity Assay Kit (Abisense, Moscow, Russia)) was added in culture medium. The cells were incubated for 4 h at 37 °C and 5% CO_2_. The fluorescence of the resazurin solution incubated in wells without cells was used as a baseline. Measurements were performed using an INNO-X multimode spectrophotometer at an excitation/emission wavelength of 535/585 nm.

### 2.5. Senescence Marker Assessment

#### 2.5.1. Senescence-Associated β-Galactosidase Staining

Staining of cells for senescence-associated β-galactosidase (SA-β-gal) to identify senescent cells was performed using the commercial Aging Cell β-galactosidase Staining Kit (Servicebio, Wuhan, China) according to the manufacturer’s instructions. Briefly, cells were fixed at room temperature for 15 min and washed three times with phosphate-buffered saline (PBS). Each well then received 1 mL of the β-galactosidase staining working solution, and the cells were incubated overnight at 37 °C. To assess SA-β-gal activity in cell culture, ImageJ software version 1.53 was used for the automatic analysis of SA-β-gal-stained cell images.

#### 2.5.2. Analysis of SASP Factor Secretion

For SASP assessment, the concentration of the following factors was measured in samples of culture fluid: IL-1β, IL-6, IL-8, MCP-1 (CCL2), and TNF-α by enzyme-linked immunosorbent assays using commercial kits Human IL-1 beta/IL-1F2 DuoSet ELISA, Human IL-6 DuoSet ELISA, Human IL-8 DuoSet ELISA, Human CCL2/MCP-1 DuoSet ELISA, and Human TNF-alpha DuoSet ELISA (R&D Systems, Minneapolis, MN, USA) according to the manufacturer’s instructions.

#### 2.5.3. Cell Cycle Arrest and DNA Damage Response Marker Measurement

The concentration of p21, p16, and p53 proteins were measured as markers of cell cycle arrest and DNA damage response in cell lysate by enzyme-linked immunosorbent assays using commercial kits Human p21 ELISA Kit ab214658, Human CDKN2A/p16INK4a ELISA Kit ab227903, and Human p53 (phospho S392) ELISA Kit ab322370 (Abcam Ltd., Waltham, MA, USA) according to the manufacturer’s instructions.

### 2.6. Evaluation of the Senolytic Efficacy of the Natural Fisetin Preparation 

**Figure 3:**
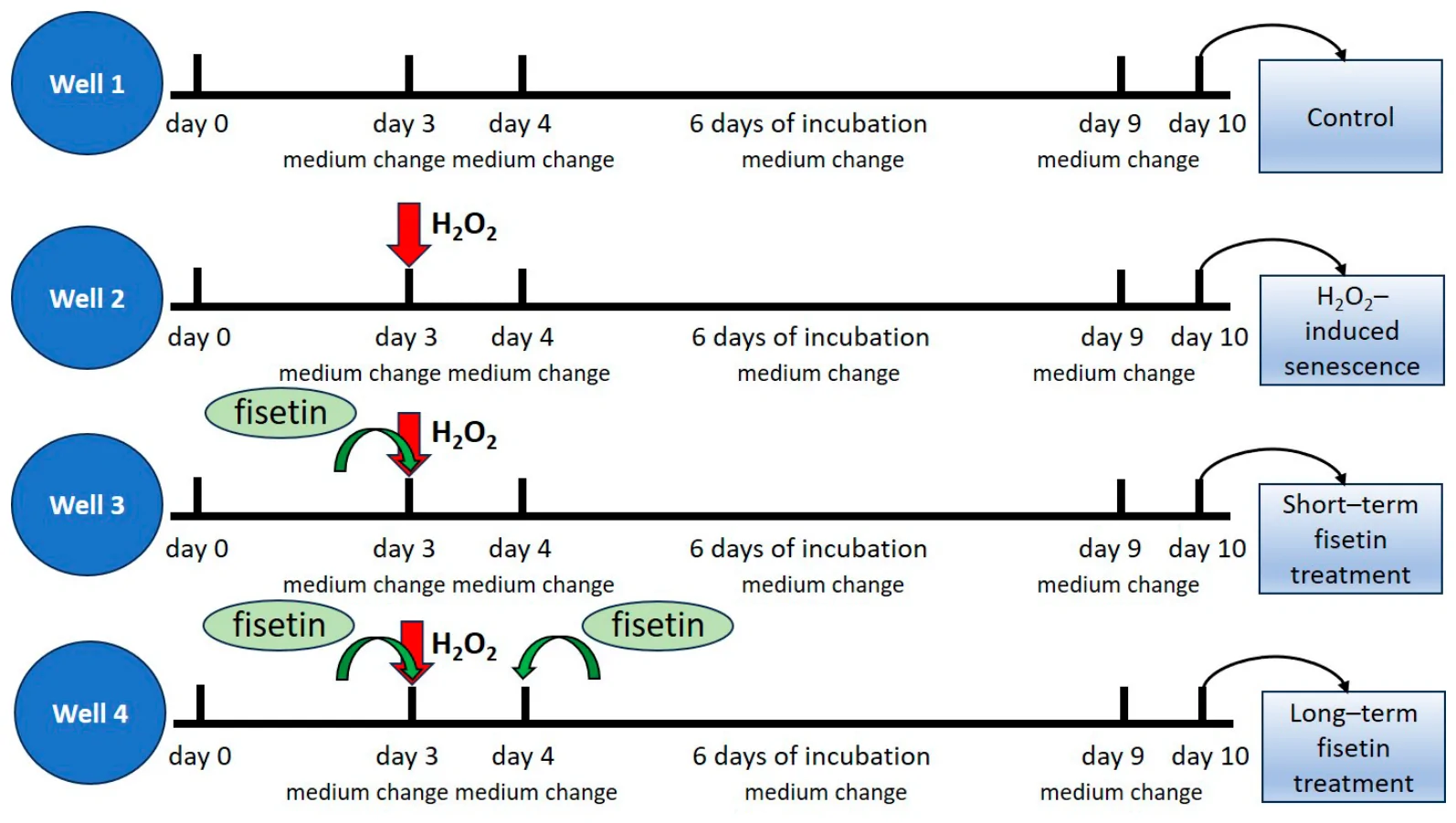
Scheme for assessing the senolytic efficacy of fisetin in the model of stress-induced cellular senescence of AD-MSCs.

The senolytic efficacy of a fisetin preparation (100 mg capsules; GraFLab, Moscow, Russia) was assessed in a model of stress-induced cellular senescence in AD-MSCs using the scheme shown in Figure 3. The preparation used in this study contained no excipients. Fisetin was dissolved in dimethyl sulfoxide (DMSO) and then diluted with culture medium to the required concentration; the final DMSO concentration in the medium did not exceed 0.001%. A fisetin solution at final concentrations of 0–300 μM was used to analyze dose-dependent efficacy and cytotoxicity. Cell viability was assessed using a colorimetric method for determining cell metabolic activity and cytotoxicity—namely a resazurin test. A fisetin solution at a final concentration of 50 μM was used for subsequent experiments.

### 2.7. Statistics

Statistical analysis of the data was performed using SPSS 27.0 (IBM Corp., Armonk, NY, USA). All experiments were conducted in 8 biological replicates (8 study participants). For each study participant, measurements were performed in three technical replicates and averaged prior to statistical analysis. Normality of distribution was assessed using the Shapiro–Wilk test. For all analyzed variables, the hypothesis of normal distribution was not rejected (*p* > 0.05). Student’s *t*-test was used to assess the statistical significance of differences between groups. Due to the exploratory and hypothesis-generating design of this study, no adjustment for multiple comparisons was performed. Data are presented as mean and standard deviation (mean (SD)). A difference in mean values at *p* < 0.05 was considered statistically significant.

## 3. Results

### 3.1. Replicative Cellular Aging Model

In the replicative cellular aging model, senescent markers of cultured AD-MSCs were assessed during long-term culturing. For this purpose, the proportion of cells positively stained for SA-β-gal (SA-β-gal+), the levels of secretion of SASP factors IL-1β, TNF-α, and MCP-1, and the concentration of cell cycle arrest markers p16, p21, and p53 were determined at the second passage of AD-MSCs (day 10 of cultivation), sixth passage (day 42 of cultivation), twelfth passage (day 95 of cultivation), and eighteenth passage (day 140 of cultivation).

**Figure 4:**
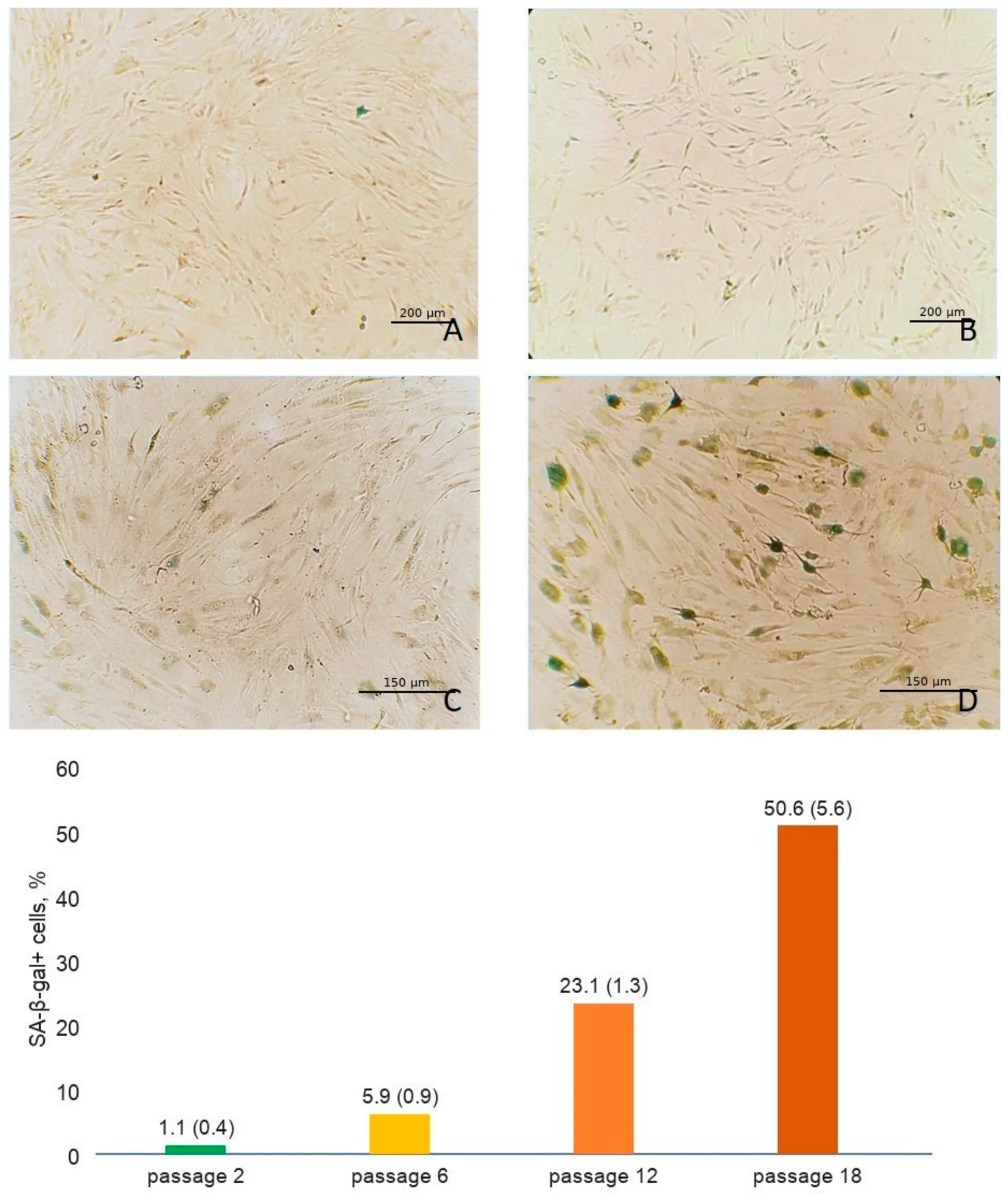
SA-β-gal staining and quantitative analysis of AD-MSCs in the model of replicative cellular aging, ×20/×40. (**A**) AD-MSCs at passage 2; (**B**) AD-MSCs at passage 6; (**C**) AD-MSCs at passage 12; (**D**) AD-MSCs at passage 18.

A significant increase in SA-β-gal+ cells was observed at passage 6 of AD-MSCs compared to the cell culture at passage 2, *p* = 0.001. The tendency toward an increase in the number of SA-β-gal+ cells persisted throughout the entire culturing period; the difference was significant at passages 12 and 18, *p* < 0.001. Images of cultured AD-MSCs stained for SA-β-gal are shown in Figure 4.

When assessing the secretion of SASP factors in culture fluid samples, it was revealed that the level of IL-1β secretion by cultured cells significantly increased at passages 12 and 18 compared to passage 2, *p* < 0.001 and *p* < 0.05, respectively, the level of TNF-α did not change significantly, and the level of MCP-1 increased insignificantly at passage 12 and statistically significantly at passage 18, *p* < 0.05. The data are presented in Table 1.

Table 2 presents the results of measuring the concentration of cell cycle arrest markers in the lysate of AD-MSCs. It was shown that the level of p16 significantly increased at passage 18 compared to passage 2; the levels of p21 and p53 significantly increased at passages 12 and 18 compared to passage 2, *p* < 0.05.

### 3.2. Stress-Induced Cellular Aging Model

**Figure 5:**
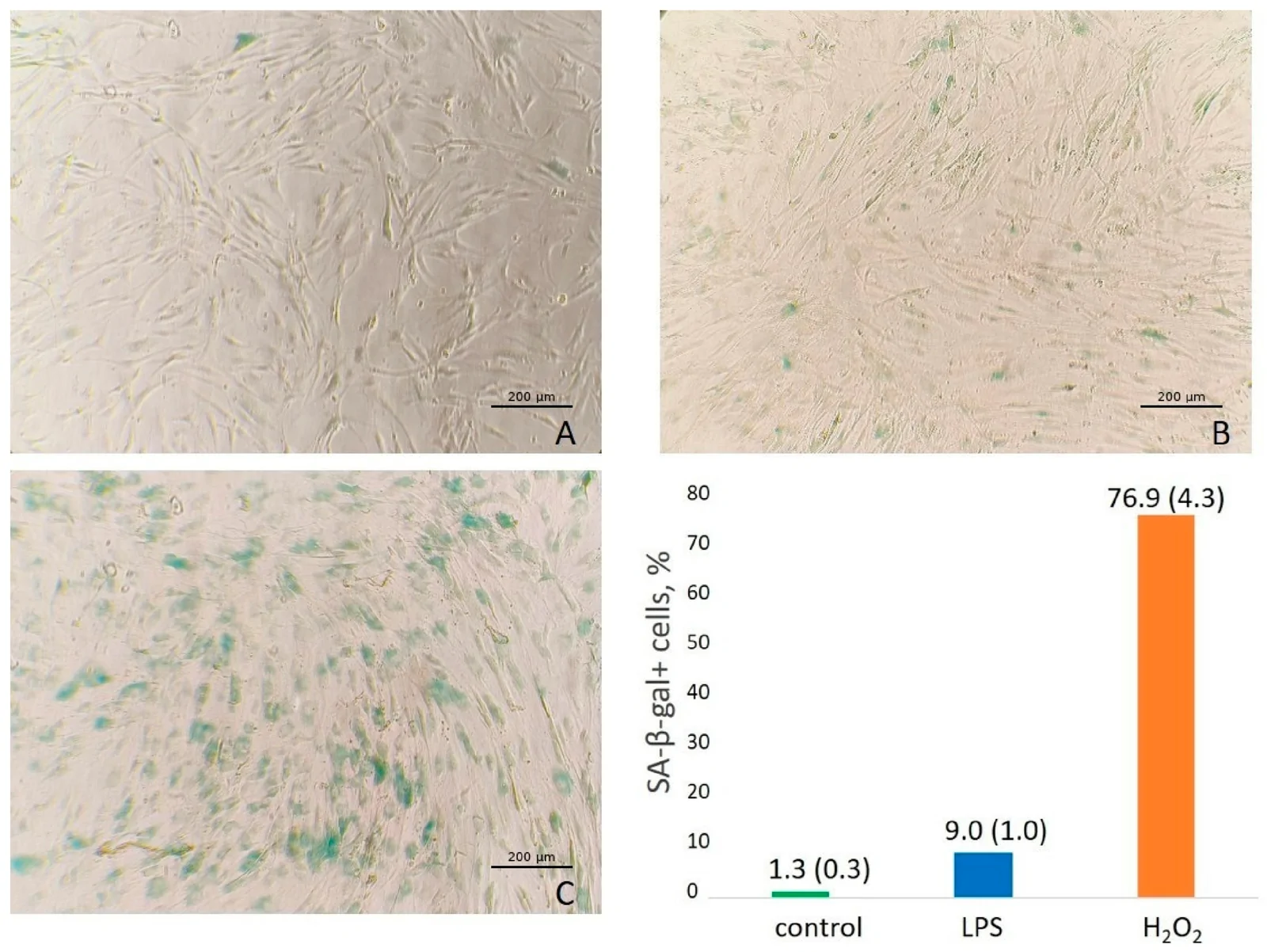
SA-β-gal staining of AD-MSCs in the model of stress-induced cellular aging, ×20. (**A**) Non-stimulated AD-MSCs; (**B**) AD-MSCs with LPS-induced senescence; (**C**) AD-MSCs with H_2_O_2_-induced senescence.

Stress-induced cellular aging was studied in AD-MSC cultures at passage 3 when stimulating senescence by addition of LPS and H_2_O_2_ to the culture medium for 24 h. The proportion of SA-β-gal+ cells, the secretion of SASP factors, and the concentration of cell cycle arrest markers p16, p21, and p53 were assessed. Figure 5 shows images of the AD-MSCs stained for SA-β-gal. It was shown that the proportion of SA-β-gal+ cells significantly increased after stress induction compared to the control. The proportion of SA-β-gal+ cells was 1.3 (0.3)% in control non-stimulated cells, 9.0 (1.0)% in LPS-stimulated cells (*p* < 0.001 compared to the control), and 76.9 (4.3)% in H_2_O_2_-stimulated cells (*p* < 0.001 compared to the control).

The stimulation of cellular senescence with LPS and H_2_O_2_ caused a significant increase in the secretion of the SASP factors MCP-1 and IL-6, as well as TNF-α upon stimulation with LPS; the data are presented in Table 3. In addition, the stimulation of cellular senescence with H_2_O_2_ led to a significant increase in the concentration of cell cycle arrest markers in the cell lysate, while stimulation with LPS did not cause significant changes; the data are presented in Table 4.

The increase in the proportion of SA-β-gal+ cells was significantly more pronounced in the H_2_O_2_-stimulated cells compared to LPS-stimulated cells. Both methods of stimulating cellular senescence demonstrated comparable efficacy in terms of SASP factor secretion and the concentration of cell cycle arrest markers. Therefore, H_2_O_2_ was used for further experiments as the most effective inducer of cellular senescence in AD-MSCs based on SA-β-gal staining data.

### 3.3. Results of the Study of Fisetin Senolytic Efficacy in the Model of Stress-Induced Cellular Aging of AD-MSCs

**Figure 6:**
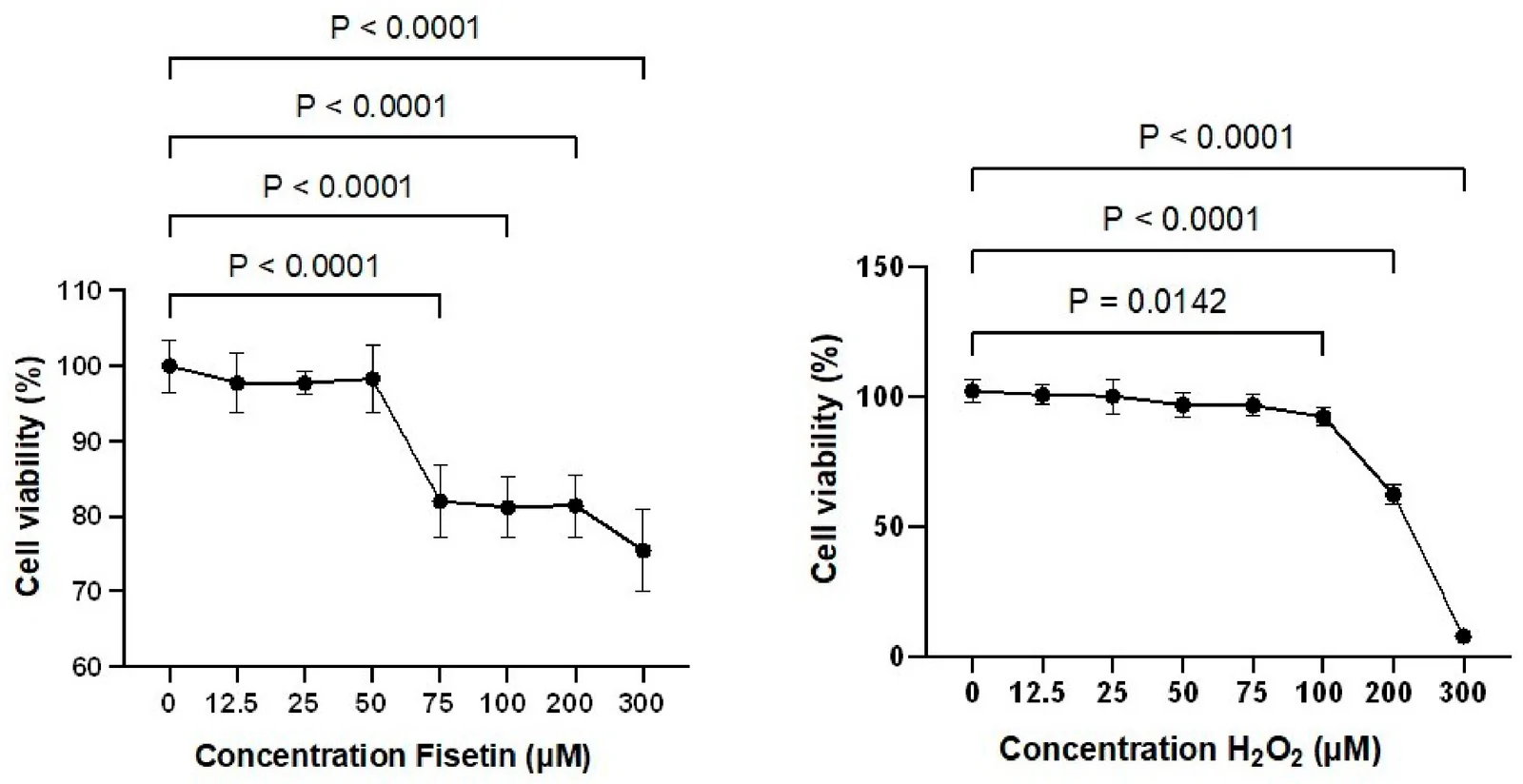
Analysis of AD-MSC viability after H_2_O_2_ and fisetin treatment.

When developing the model of stress-induced senescence of AD-MSCs, the most pronounced increase in senescent markers was demonstrated during H_2_O_2_ stimulation; therefore, this model was considered the most relevant for subsequent experiments aiming to evaluate the senolytic efficacy of a natural preparation based on natural polyphenols such as fisetin. To select optimal conditions, AD-MSCs were treated with different concentrations of H_2_O_2_ and fisetin (0–300 μM) for 24 h, and cell viability was measured (Figure 6).

**Figure 7:**
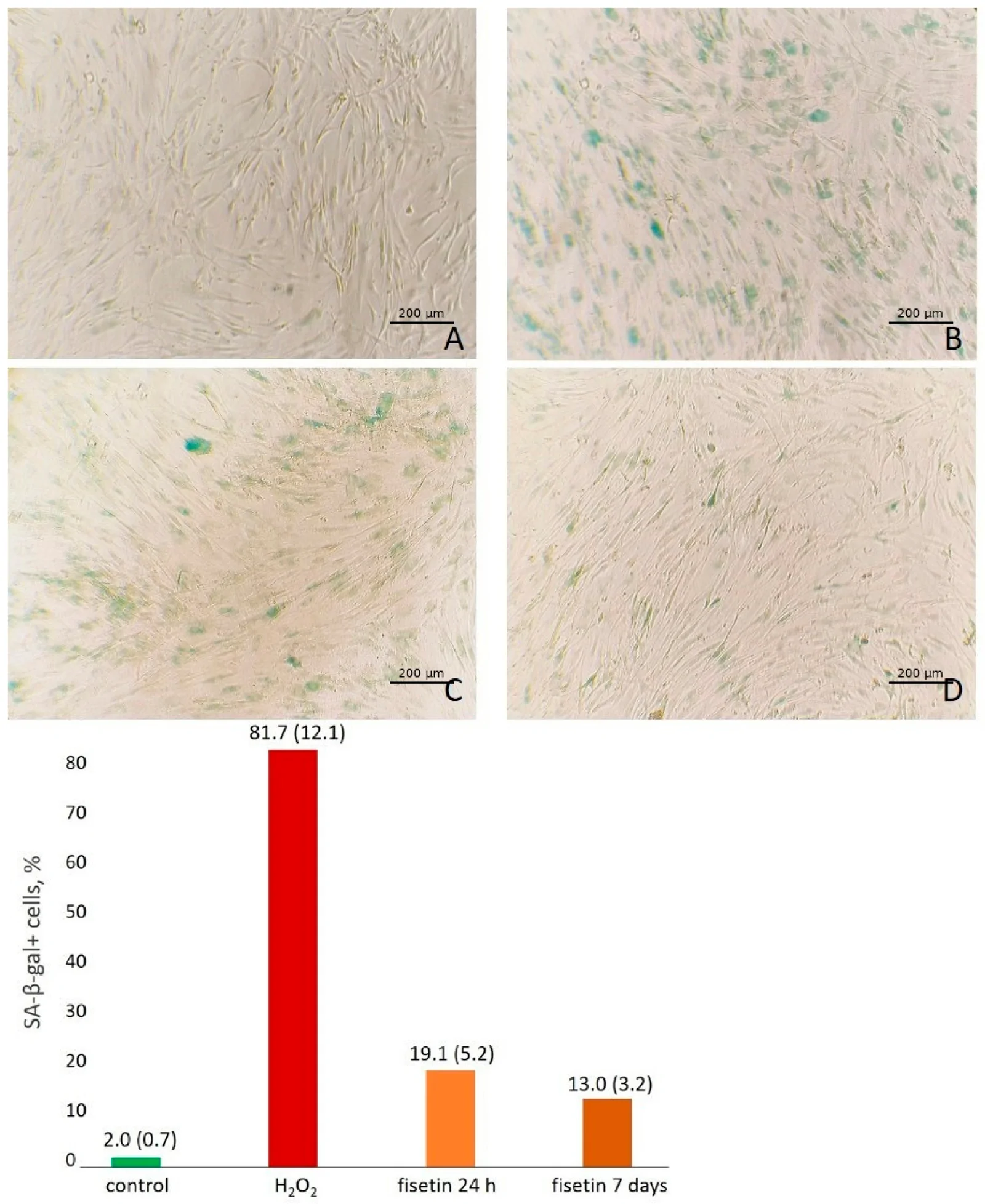
Effect of fisetin on the number of SA-β-gal+ cells in the stress-induced aging model of AD-MSCs, ×20. (**A**) Non-stimulated AD-MSCs without fisetin treatment; (**B**) AD-MSCs with H_2_O_2_-induced senescence without fisetin treatment; (**C**) AD-MSCs with H_2_O_2_-induced senescence after 24 h of fisetin treatment; (**D**) AD-MSCs with H_2_O_2_-induced senescence after 7 days of fisetin treatment.

It was shown in the model of stress-induced cellular senescence of AD-MSCs that the proportion of SA-β-gal+ cells significantly increased during H_2_O_2_ stimulation compared to the control cells without stimulation, *p* < 0.001. The incubation of H_2_O_2_-stimulated cells with fisetin led to a statistically significant decrease in the proportion of SA-β-gal+ cells both after 24 h treatment and after long-term incubation for 7 days, *p* < 0.001, compared to H_2_O_2_-stimulated cells without fisetin; the data are presented in Figure 7.

Table 5 presents the results of evaluating the effect of fisetin on SASP factor secretion in the stress-induced aging model of AD-MSCs. It was shown that during the first day of incubation with fisetin, H_2_O_2_-stimulated secretion of MCP-1 statistically significantly decreased; the long-term incubation of cultured AD-MSCs with fisetin led to the most pronounced decrease in H_2_O_2_-stimulated MCP-1 secretion. H_2_O_2_-stimulated secretion of IL-8 significantly decreased after 24 h of incubation with fisetin but significantly increased after 7 days of incubation both in cells with and without fisetin treatment. Secretion of IL-1β statistically significantly decreased after 7 days of incubation with fisetin. No significant changes were observed for TNF-α and IL-6 secretion.

The assessment results of cell cycle arrest marker concentration in AD-MSCs are presented in Table 6. It was shown that H_2_O_2_ stimulation led to a statistically significant increase in p16, p21, and p53 concentrations. Incubation with fisetin led to an insignificant decrease in the concentration of p16 after short-term as well as after long-term treatment; an insignificant increase in p53 after 24 h and after 7 days of incubation; and a significant increase in p21 both after 24 h of exposure and during prolonged stimulation for 7 days, *p* < 0.05, compared to the control and H_2_O_2_-stimulated cells in all cases.

## 4. Discussion

In the current study, AD-MSCs and adipocytes differentiated from AD-MSCs were used to develop a model of cellular senescence. The effects of bacterial LPS and H_2_O_2_ were studied as stimulators of cellular senescence. In published data from previous studies, the LPS-induced cellular senescence model is widely used. It has been shown that in a number of cellular models, LPS has a significant effect on the secretion of SASP factors, the expression of cell cycle arrest markers, and staining for SA-β-gal and other senescence markers; however, these studies did not evaluate the LPS induction of senescence in AD-MSCs [17]. H_2_O_2_ is an inducer of oxidative stress, one of the key factors of aging, and is also used in experimental studies to investigate cellular senescence [18]. The H_2_O_2_-induced AD-MSC aging model has been used in a number of studies aimed at studying the pathogenesis of MSC aging and assessing the efficacy and mechanisms of the gerontoprotective action of some therapeutic agents, but the exposure time and dosage of H_2_O_2_ in different experiments vary significantly [19,20,21]. Based on the current study results, H_2_O_2_ was selected for further experiments as the most effective cellular senescence inducer, since it increased the content of SA-β-gal+ cells with comparable activity to LPS in relation to cell cycle arrest markers and SASP factors.

In the present study, SASP factors, cell cycle arrest markers p16, p21, and p53, and SA-β-gal staining were used as senescence markers. Cellular senescence is characterized by cell cycle arrest in the G1 or possibly G2 phase, which prevents the proliferation of damaged cells. In contrast, the G0 phase is characterized by cellular quiescence, with a reversible state of growth arrest [22]. The p53 protein is a regulator of cell growth and division and is involved in the induction of senescence as a mechanism preventing the oncotransformation of cells. In response to stress or damage, activation of the p53 signaling pathway triggers various reactions, including cell cycle arrest, DNA repair, or apoptosis in order to prevent the proliferation of damaged cells [23]. Persistent DNA damage with severe telomere shortening recruits DNA damage sensor proteins, causing stabilization and activation of the p53 suppressor protein. Induction of the p53/p21WAF1/CIP1 pathway leads to irreversible cell cycle arrest and can occur independently of telomere shortening resulting from premature aging under the influence of oxidative stress, as well as epigenetic modifications, DNA damage, and other factors [24]. p21WAF1/CIP1 is a p53 effector protein involved in the regulation of the cell cycle and cell growth. p16INK4A and p21WAF1/CIP1 are inhibitors of cyclin-dependent CDK kinases, CDK4/6, and CDK2 and act as negative regulators of cell cycle progression [25]. Long-term expression of any of the cell cycle arrest proteins is sufficient to induce senescence. Stress-induced senescence is accompanied by DNA damage and activates DNA damage response. Persistent DNA damage signaling leads to the production of SASP factors [26]. In the present study, the secretion of the following SASP factors was assessed in the development of cellular senescence models to evaluate the effectiveness of fisetin: cytokines IL-1β, TNF-α, IL-6, and chemokines MCP-1 and IL-8.

A specific form of β-galactosidase, SA-β-gal, is widely used as a biochemical marker to identify senescent cells. Increased SA-β-gal activity in senescent cells is due to the leakage of lysosomal β-galactosidase into the cytoplasm as the lysosomal membrane becomes more permeable [27]. SA-β-gal is the most widely used marker of cellular senescence. In particular, it was shown in a culture of rat bone marrow-derived MSCs that incubation with a substrate-lowering pH in lysosomes resulted in a decrease in SA-β-gal activity, along with reduced expression of p21 and elevated expression of lysosome-associated membrane proteins 1 and 2 [28]. At the same time, SA-β-gal activity does not determine the mechanism of cellular senescence and can be considered only as a biochemical marker, since enzyme inhibition is not able to prevent the aging process. In this study, cell staining for SA-β-gal was the main criterion for choosing the AD-MSC culture as a basis for developing a model of cellular aging, since LPS and H_2_O_2_ stimulation of AT-MSC senescence showed that with similar activity of both inducers in relation to SASP factors and markers of cell cycle arrest, stimulation with H_2_O_2_ led to a significantly greater increase in the number of SA-β-gal+ cells in culture compared to LPS, 76.9 (4.3)% vs. 9.0 (1.0)%, respectively, *p* < 0.001.

The present study in the H_2_O_2_-induced senescence model of AD-MSCs showed that incubation with fisetin resulted in a statistically significant decrease in SA-β-gal+ cells after a short-term 24 h exposure, as well as after long-term incubation for 7 days, compared to H_2_O_2_-stimulated cells without fisetin treatment. In particular, the proportion of SA-β-gal+ cells was 2.0 (0.7)% in non-stimulated AD-MSCs, 81.7 (12.1)% in H_2_O_2_-induced AD-MSCs without fisetin, and 19.1 (5.2)% and 13.0 (3.2)% after short-term and long-term fisetin treatment, respectively, demonstrating the high efficiency of fisetin against SA-β-gal+, one of the key markers of cellular senescence. However, when assessing the effect of fisetin on the secretion of SASP factors, conflicting data were obtained. It was shown that the addition of fisetin led to a statistically significant decrease in the secretion of the chemokine MCP-1 and an increase in the secretion of IL-8, especially during long-term treatment. Furthermore, incubation of cells with fisetin resulted in increased concentrations of cell cycle arrest markers p53 and p21. However, the increase in p53 and p21 observed after fisetin treatment should not be regarded as direct pro-senescence activity. It was shown in previous studies that fisetin’s senolytic activity is due to the activation of apoptosis [29,30]. However, the current study did not examine the effects of fisetin on apoptotic pathways in the stress-induced aging model of MSCs, which is a limitation of the study. Recent studies demonstrated that fisetin selectively induces apoptosis in cellular models based on several types of senescent cells such as HUVECs and fibroblasts targeting multiple pathways, including PI3K/AKT and Bcl-2 family members [29]. The other studies demonstrated that fisetin influences key cell signaling pathways related to inflammation, angiogenesis, and transcription factors and induces activation of apoptotic cellular pathways accompanied by increased levels of p53 and p21 [30]. Fisetin has demonstrated senolytic activity in animal models, including reductions in senescent-cell burden and SASP-associated inflammation in aged mice [31,32]. Clinical trials are currently underway to study the efficacy of fisetin in chronic diseases associated with age, in particular, in frailty, type 2 diabetes mellitus, and osteoarthritis; nevertheless, its clinical efficacy remains to be established [33].

Fisetin is a natural flavonoid that possesses senolytic effects targeting anti-apoptotic pathways in senescent cells. The effects of fisetin on cellular senescence in AD-MSCs have not been well studied. Only one study has shown that fisetin effectively influences the expression of cellular senescence markers in a replicative aging model of AD-MSCs; in particular, it reduces the content of reactive oxygen species, SA-β-gal activity, and the number of senescence-associated heterochromatin foci [34]. A number of studies in aging models using murine and human fibroblasts, as well as in mice with progeroid syndrome, have shown that fisetin treatment reduces aging markers in many tissues in both young and old mice, reduces inflammation and oxidative stress, and improves tissue homeostasis [35]. Fisetin has previously been shown to significantly reduce the number of SA-β-gal+ cells and decrease the secretion of SASP factors in cultured human senescent skin fibroblasts and in skin samples obtained by subcutaneous transplantation of whole-skin grafts from elderly individuals into nude mice [36].

Thus, in the current study, a model of H_2_O_2_-induced cellular senescence of AD-MSCs was developed, and the efficacy of a natural preparation based on the natural polyphenol fisetin was studied. The developed cellular model is promising for practical use as a platform for screening senolytic and geroprotective compounds, suggesting their translation into clinical practice for the prevention of age-associated diseases and the maintenance of tissue function. Fisetin treatment was associated with a decrease in SA-β-gal positivity and selected SASP factors; however, the concurrent increase in p53 and p21 warrants further mechanistic investigation. Therefore, fisetin should be further evaluated in aging models.

A limitation of the present study was the use of a narrow range of cellular aging markers; in particular, parameters such as the presence of heterochromatin foci associated with aging, γH2AX—a marker of DNA damage associated with aging—lipofuscin, telomere length, and telomerase activity were not assessed [37]. Another limitation of the study was that AD-MSC cultures were obtained only from one group of obese patients aged 37.0 (3.3) years. Comparison of AT-MSC aging parameters in patients of different age groups with normal body mass index and different stages of obesity is an important area of research. A more in-depth study of the mechanisms of AD-MSC senescence is also a prospect for further research, such as an investigation of the transcriptome profile for the analysis of signaling pathways involved in cellular aging, oxidative stress, mitochondrial dysfunction, and autophagy in the pathogenesis of the cellular aging of AD-MSCs and other adipose tissue cells, as well as a study of the senolytic efficacy of various therapeutic agents using the developed cellular model of aging, as well as in animal models of aging and in clinical trials in patients with obesity and concomitant age-associated diseases.

## 5. Conclusions

MSCs play an important role in maintaining body homeostasis, promoting tissue regeneration and the balance of inflammatory responses. This study developed a model of H_2_O_2_-induced cellular senescence in AD-MSCs, which can be used to evaluate the potential of preparations and natural products against cellular senescence in AD-MSCs to develop new senotherapeutic strategies, which is a priority area of research in the field of preventive medicine for healthy longevity. Fisetin warrants further investigation as a potential senotherapeutic agent targeting AD-MSC senescence.

## Figures and Tables

**Table 1:** SASP factor secretion by AD-MSCs in the model of replicative aging.

Concentration, pg/mL	IL-1β	TNF-α	MCP-1
passage 2	58.1 (6.4)	67.4 (11.3)	811.2 (17.9)
passage 6	50.5 (3.3)	69.3 (8.9)	798.6 (42.1)
passage 12	122.3 (7.2) *	78.5 (6.2)	1156.8 (134.0)
passage 18	98.0 (8.6) *	95.4 (21.1)	1203.3 (54.1) *

*, significant difference in comparison with passage 2, *p* < 0.05.

**Table 2:** Cell cycle arrest markers in the model of replicative aging of AD-MSCs.

Concentration, pg/mL	p16	p21	p53
passage 2	34.5 (8.3)	10,644.0 (482.8)	81.0 (8.4)
passage 6	38.3 (6.2)	10,054.3 (371.5)	84.8 (11.4)
passage 12	41.6 (11.8)	13,782.7 (562.8) *	97.2 (15.5) *
passage 18	68.7 (5.0) *	14,205.8 (114.9) *	106.7 (13.9) *

*, significant difference in comparison with passage 2, *p* < 0.05.

**Table 3:** SASP factor secretion by AD-MSCs in the model of stress-induced aging.

Concentration, pg/mL	IL-1β	TNF-α	MCP-1	IL-6
Non-stimulated	54.2 (3.1)	87.2 (12.2)	785.1 (24.8)	246.6 (11.7)
LPS	58.2 (4.5)	145.1 (12.6) *	1496.0 (119.22) *	459.0 (34.1) *
H_2_O_2_	53.7 (5.5)	92.6 (17.9)	1119.9 (59.3) *	298.8 (8.2) *

*, significant difference in comparison with non-stimulated cells, *p* < 0.05.

**Table 4:** Cell cycle arrest markers in the model of stress-induced aging of AD-MSCs.

Concentration, pg/mL	p53	p16	p21
Non-stimulated	76.5 (0.9)	34.6 (2.9)	12,837.2 (500.2)
LPS	58.2 (27.0)	29.5 (7.6)	12,492.4 (248.5)
H_2_O_2_	85.2 (4.2) *	32.4 (0.6)	14,748.9 (526.2) *

*, significant difference in comparison with non-stimulated cells, *p* < 0.05.

**Table 5:** The effect of fisetin treatment on SASP factor secretion by AD-MSCs in the stress-induced aging model.

**IL-1β**		
Concentration, pg/mL	measurement at 24 h of incubation	measurement after 7 days of incubation
control	56.7 (7.1)	46.6 (8.1)
H_2_O_2_	69.8 (17.5)	50.8 (6.2)
H_2_O_2_ + fisetin, 24 h treatment	67.0 (10.2)	59.7 (14.8)
H_2_O_2_ + fisetin, 7 days treatment	47.4 (10.1)	40.4 (9.1)
p (control vs. H_2_O_2_)	0.091	0.518
p (H_2_O_2_ vs. H_2_O_2_ + fisetin 24 h)	0.553	0.003
p (H_2_O_2_ vs. H_2_O_2_ + fisetin 7 days)	0.019	0.022
**MCP-1**		
Concentration, pg/mL	measurement after 24 h of incubation	measurement after 7 days of incubation
control	748.6 (23.9)	1382.2 (40.0)
H_2_O_2_	1286.2 (113.7)	1782.1 (18.8)
H_2_O_2_ + fisetin, 24 h treatment	910.4 (1.4)	1613.5 (33.0)
H_2_O_2_ + fisetin, 7 days treatment	828.4 (4.8)	1251.8 (49.4)
p (control vs. H_2_O_2_)	0.078	0.001
p (H_2_O_2_ vs. H_2_O_2_ + fisetin 24 h)	0.001	0.059
p (H_2_O_2_ vs. H_2_O_2_ + fisetin 7 days)	0.011	<0.001
**IL-8**		
Concentration, pg/mL	measurement after 24 h of incubation	measurement after 7 days of incubation
control	131.0 (5.8)	104.4 (2.4)
H_2_O_2_	133.9 (2.4)	131.5 (0.7)
H_2_O_2_ + fisetin, 24 h treatment	85.3 (0.6)	166.7 (2.1)
H_2_O_2_ + fisetin, 7 days treatment	93.4 (5.2)	237.3 (4.9)
p (control vs. H_2_O_2_)	0.753	0.001
p (H_2_O_2_ vs. H_2_O_2_ + fisetin 24 h)	0.001	<0.001
p (H_2_O_2_ vs. H_2_O_2_ + fisetin 7 days)	0.020	<0.001

**Table 6:** The effect of fisetin treatment on cell cycle arrest markers in AD-MSCs in the stress-induced aging model.

Concentration, pg/mL	p53	p16	p21
control	54.3 (6.2)	31.0 (0.9)	11,329.9 (413.1)
H_2_O_2_	101.7 (13.9) *	45.2 (2.2) *	16,612.2 (438.7) *
H_2_O_2_ + fisetin, 24 h	126.9 (18.3)	31.7 (2.6)	23,151.4 (543.4) **
H_2_O_2_ + fisetin, 7 days	160.0 (46.6)	20.6 (6.1)	22,052.1 (586.0) **

*, significant difference in comparison with control cells, *p* < 0.05; **, significant difference in comparison with H_2_O_2_-stimulated cells, *p* < 0.05.

## Data Availability

The data that support the findings of this study are available from the corresponding author, Ivan V. Zhivodernikov, upon reasonable request. The data are not publicly available due to privacy and ethical restrictions.

## References

[1] Sebaa R. (2026). Exploring Adipose Tissue Complexity Through Omics Approaches: Implications for Health and Disease. Cells.

[2] Park S., Shimokawa I. (2024). Influence of Adipokines on Metabolic Dysfunction and Aging. Biomedicines.

[3] Widjaja S.S., Rusdiana R., Helvi T.M., Simanullang R.H., Jayalie V.F., Amelia R., Arisa J. (2024). Finding a Link between Obesity and Senescence: A Systematic Review and Meta-Analysis. Iran. J. Public Health.

[4] Conley S.M., Hickson L.J., Kellogg T.A., McKenzie T., Heimbach J.K., Taner T., Tang H., Jordan K.L., Saadiq I.M., Woollard J.R. (2020). Human Obesity Induces Dysfunction and Early Senescence in Adipose Tissue-Derived Mesenchymal Stromal/Stem Cells. Front. Cell Dev. Biol..

[5] Zhang Y., Jiang Y., Yang X., Huang Y., Pan A., Liao Y. (2024). Adipose tissue senescence: Biological changes, hallmarks and therapeutic approaches. Mech. Ageing Dev..

[6] Voynova E., Kulebyakin K., Grigorieva O., Novoseletskaya E., Basalova N., Alexandrushkina N., Arbatskiy M., Vigovskiy M., Sorokina A., Zinoveva A. (2022). Declined adipogenic potential of senescent MSCs due to shift in insulin signaling and altered exosome cargo. Front. Cell Dev. Biol..

[7] Rytel M., Chodkowska-Michalowska M., Sulejczak D., Krześniak N., Zychowicz M., Sarnowska A. (2025). Enhancing the clinical potential: Quality assessment of adipose tissue-derived Mesenchymal Stem/Stromal Cells for therapeutic use. Adv. Med. Sci..

[8] Yu S., Klomjit N., Jiang K., Zhu X.Y., Ferguson C.M., Conley S.M., Obeidat Y., Kellogg T.A., McKenzie T., Heimbach J.K. (2023). Human Obesity Attenuates Cardioprotection Conferred by Adipose Tissue-Derived Mesenchymal Stem/Stromal Cells. J. Cardiovasc. Transl. Res..

[9] Dangerfield J.A., Metzner C. (2026). Collecting Eggs, Not Killing Chickens: Why Stem Cell Secretome and Exosomes Are Redefining Regenerative Medicine for Healthspan Extension. Biomedicines.

[10] Zheng L., He S., Wang H., Li J., Liu Y., Liu S. (2024). Targeting Cellular Senescence in Aging and Age-Related Diseases: Challenges, Considerations, and the Emerging Role of Senolytic and Senomorphic Therapies. Aging Dis..

[11] Kang E., Kang C., Lee Y.S., Lee S.J.V. (2024). Brief guide to senescence assays using cultured mammalian cells. Mol. Cells.

[12] Yan J., Chen S., Yi Z., Zhao R., Zhu J., Ding S., Wu J. (2024). The role of p21 in cellular senescence and aging-related diseases. Mol. Cells.

[13] Samiminemati A., Shahzaib M., Moriello C., Alessio N., Aprile D., Squillaro T., Di Bernardo G., Galderisi U. (2025). Mesenchymal Stromal Cell (MSC) Isolation and Induction of Acute and Replicative Senescence. Methods Mol. Biol..

[14] Al-Azab M., Safi M., Idiiatullina E., Al-Shaebi F., Zaky M.Y. (2022). Aging of mesenchymal stem cell: Machinery, markers, and strategies of fighting. Cell. Mol. Biol. Lett..

[15] Kashyap D., Garg V.K., Tuli H.S., Yerer M.B., Sak K., Sharma A.K., Kumar M., Aggarwal V., Sandhu S.S. (2019). Fisetin and Quercetin: Promising Flavonoids with Chemopreventive Potential. Biomolecules.

[16] Dominici M., Le Blanc K., Mueller I., Slaper-Cortenbach I., Marini F., Krause D., Deans R., Keating A., Prockop D.J., Horwitz E. (2006). Minimal criteria for defining multipotent mesenchymal stromal cells. The International Society for Cellular Therapy position statement. Cytotherapy.

[17] Suzuki K., Susaki E.A., Nagaoka I. (2022). Lipopolysaccharides and Cellular Senescence: Involvement in Atherosclerosis. Int. J. Mol. Sci..

[18] Veronesi F., Contartese D., Di Sarno L., Borsari V., Fini M., Giavaresi G. (2023). In Vitro Models of Cell Senescence: A Systematic Review on Musculoskeletal Tissues and Cells. Int. J. Mol. Sci..

[19] Lee H., Massaro M., Abdelfattah N., Baudo G., Liu H., Yun K., Blanco E. (2025). Nuclear respiratory factor-1 (NRF1) induction as a powerful strategy to deter mitochondrial dysfunction and senescence in mesenchymal stem cells. Aging Cell.

[20] Fang Y., Mou C., Yu X., Zhang J., Zhu W., Zhou C., Jiang B., Chen Y. (2026). Ginsenoside Rg1 delays the senescence of adipose-derived stem cells: Network pharmacology and experimental validation. Hereditas.

[21] Shang L., Li X., Ding X., Liu G., Pan Z., Chen X., Wang Y., Li B., Wang T., Zhao R.C. (2024). S-Adenosyl-l-Methionine Alleviates the Senescence of MSCs Through the PI3K/AKT/FOXO3a Signaling Pathway. Stem Cells.

[22] Sikora E., Bielak-Zmijewska A., Mosieniak G. (2021). A common signature of cellular senescence; does it exist?. Ageing Res. Rev..

[23] Gao H., Nepovimova E., Heger Z., Valko M., Wu Q., Kuca K., Adam V. (2023). Role of Hypoxia in Cellular Senescence. Pharmacol. Res..

[24] Kumari R., Jat P. (2021). Mechanisms of Cellular Senescence: Cell Cycle Arrest and Senescence Associated Secretory Phenotype. Front. Cell Dev. Biol..

[25] Manu K.A., Cao P.H.A., Chai T.F., Casey P.J., Wang M. (2019). P21cip1/Waf1 Coordinate Autophagy, Proliferation and Apoptosis in Response to Metabolic Stress. Cancers.

[26] Hou J., Zheng Y., Gao C. (2023). Regulation of Cellular Senescence by Innate Immunity. Biophys. Rep..

[27] Itahana K., Campisi J., Dimri G.P. (2007). Methods to Detect Biomarkers of Cellular Senescence: The Senescence-Associated Beta-Galactosidase Assay. Methods Mol. Biol..

[28] Wang L., Han X., Qu G., Su L., Zhao B., Miao J. (2018). A PH Probe Inhibits Senescence in Mesenchymal Stem Cells. Stem Cell Res. Ther..

[29] Ozdemir S.A., Faizan M.I., Kaur G., Shaikh S.B., Ul Islam K., Rahman I. (2025). Heterogeneity of Cellular Senescence, Senotyping, and Targeting by Senolytics and Senomorphics in Lung Diseases. Int. J. Mol. Sci..

[30] Zamanian M.Y., Taheri N., Ramadan M.F., Mustafa Y.F., Alkhayyat S., Sergeevna K.N., Alsaab H.O., Hjazi A., Molavi Vasei F., Daneshvar S. (2024). A comprehensive view on the fisetin impact on colorectal cancer in animal models: Focusing on cellular and molecular mechanisms. Anim. Models Exp. Med..

[31] Tavenier J., Nehlin J.O., Houlind M.B., Rasmussen L.J., Tchkonia T., Kirkland J.L., Andersen O., Rasmussen L.J.H. (2024). Fisetin as a senotherapeutic agent: Evidence and perspectives for age-related diseases. Mech. Ageing Dev..

[32] Mahoney S.A., Venkatasubramanian R., Darrah M.A., Ludwig K.R., VanDongen N.S., Greenberg N.T., Longtine A.G., Hutton D.A., Brunt V.E., Campisi J. (2024). Intermittent supplementation with fisetin improves arterial function in old mice by decreasing cellular senescence. Aging Cell.

[33] Wissler Gerdes E.O., Misra A., Netto J.M.E., Tchkonia T., Kirkland J.L. (2021). Strategies for late phase preclinical and early clinical trials of senolytics. Mech. Ageing Dev..

[34] Mullen M., Nelson A.L., Goff A., Billings J., Kloser H., Huard C., Mitchell J., Hambright W.S., Ravuri S., Huard J. (2023). Fisetin Attenuates Cellular Senescence Accumulation During Culture Expansion of Human Adipose-Derived Stem Cells. Stem Cells.

[35] Yousefzadeh M.J., Zhu Y., McGowan S.J., Angelini L., Fuhrmann-Stroissnigg H., Xu M., Ling Y.Y., Melos K.I., Pirtskhalava T., Inman C.L. (2018). Fisetin is a senotherapeutic that extends health and lifespan. eBioMedicine.

[36] Takaya K., Asou T., Kishi K. (2024). Fisetin, a potential skin rejuvenation drug that eliminates senescent cells in the dermis. Biogerontology.

[37] Kirichenko T.V., Markina Y.V., Markin A.M., Vasilyev V.S., Hua H., Li D., Woo A.Y., Deev R.V., Eremin I.I., Kotenko K.V. (2025). Functional Features of Senescent Cells and Implications for Therapy. Int. J. Mol. Sci..

